# Localized Relapse of Primary Plasma Cell Leukaemia in the Central Nervous System 

**DOI:** 10.1155/2015/273565

**Published:** 2015-05-10

**Authors:** Christian W. Eskelund, Niels Frost Andersen

**Affiliations:** Department of Haematology, Aarhus University Hospital, Aarhus, Denmark

## Abstract

Primary plasma cell leukaemia (pPCL) is a rare and aggressive form of plasma cell malignancies with a very poor prognosis. Compared to other plasma cell malignancies the tendency to extramedullary spread is increased; however central nervous system (CNS) involvement is rare and only reported in few cases. We report the case of a 61-year-old man who was diagnosed with pPCL and achieved a complete remission after autologous stem cell transplantation but had a relapse in the CNS without systemic disease.

## 1. Introduction

Primary plasma cell leukaemia (pPCL) is a rare and aggressive disease with a prevalence of only 1–4% of all plasma cell malignancies [1–3]. It is defined by the presence of >2 ∗ 10^9^/L peripheral blood plasma cells or plasmacytosis accounting for >20% of the white cell count [1]. The prognosis of pPCL is very poor with a median overall survival (OS) of 8–11 months [1, 4]. However, the outcome of pPCL has improved by the introduction of autologous stem cell transplantation as well as novel agents like Bortezomib [1, 4–7].

Compared to multiple myeloma (MM), the presenting signs and symptoms of pPCL have a more rapid onset with higher tendency of hypermetabolic symptoms (weight loss, fevers, sweating, fatigue, and weakness) extramedullary manifestations, hypercalcaemia, renal involvement, bone marrow failure, and higher beta-2-microglobulin but rarely osteolytic bone lesions [7]. Furthermore, the presence of poor-risk cytogenetic alterations known from MM is markedly higher [1, 4, 8].

Only a few cases of central nervous system (CNS) involvement in pPCL have been published, and in these both clinical and paraclinical presentation, as well as treatment approaches and clinical courses, were very diverse [9–13]. Hence, treatment strategies rely on the experiences with CNS involvement of MM, which is estimated to involve approximately 1% of all MM cases [14, 15]. These support the administration of intrathecal chemotherapy (including Cytarabine, Methotrexate, and Methylprednisolone) and radiotherapy. Even with optimal treatment the OS after CNS involvement of MM is very short, 3–6 months [14–16].

## 2. Case

A 61-year-old—previously healthy—male was diagnosed with pPCL in October 2008. He presented 10 days earlier with flu-like symptoms and was admitted to a general hospital. Laboratory findings revealed haemoglobin 9.3 g/dL, WBC 17.4 ∗ 10^9^/L, lymphocyte count 9.7 ∗ 10^9^/L dominated by atypical cells, platelets 112 ∗ 10^9^/L, CRP 202 nmol/L, and sedimentation ratio 112 mm. The patient was referred to a haematological department.

Peripheral blood smear revealed 25–35% abnormal plasma cells, 50% in bone marrow aspirate and 80% in the bone marrow biopsy with lambda monoclonality. Plasma M-protein is 32 g/L (IgA lambda), creatinine is 106 *μ*mol/L, ionized calcium is 1.35 mmol/L, beta-2-microglobulin is 9.0 mg/L, and albumin is 439 *μ*mol/L. Urine M-protein is 0.032 g/L (lambda). Radiological skeletal survey of skull, spine, pelvis, and long bones was without osteolytic bone lesions. According to the International Staging System (ISS) of MM the patient was in stage III.

**Figure 1:**
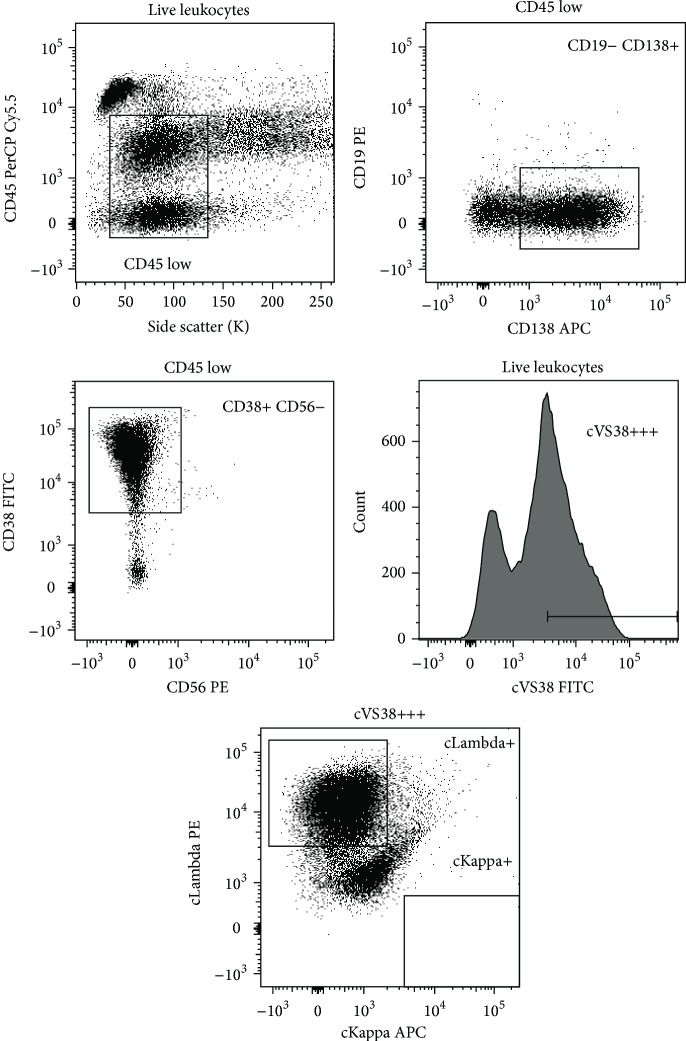
Flow cytometric data on peripheral blood at diagnosis, October 2008. The malignant clone is CD138+, CD38+, and CD56− and displays lambda clonality.

Cytogenetic analyses showed a complex, hyperdiploid clone (Karyotype 45–48,XY, +1.del(1)(p22), add(7)(q36), del(8)(q22), -10, -16,+mar1(cp20)). FISH analysis was done for del13q and IgH-rearrangement and both were negative. Unfortunately, FISH analysis for del17p was not part of our standard procedure at the time of diagnosis. Flow cytometric analyses of the blood showed 57% plasma cells (CD38high/CD45neg, CD138pos, CD56neg, and monoclonal of lambda) (Figure 1).

A final diagnosis of IgA lambda pPCL was made. The patient received 3 monthly cycles of standard VAD regimen (Vincristine, Doxorubicin, and Dexamethasone), cyclophosphamide priming with subsequent leukapheresis and proceeded to high-dose Melphalan 200 mg/m^2^ and autologous bone marrow transplantation in March 2009. Morphological and flow cytometric evaluation of blood and bone marrow showed a stringent complete remission according to the IMWG PCL response criteria [17].

In January 2011 the patient presented with lower back pain and an MRI scan revealed a tumour of 25 × 10 mm in the intraspinal space, but extramedullary at the level of L2. The tumour was removed surgically and pathological examination confirmed the diagnosis of plasmacytoma (lambda monoclonality). Examination of bone marrow and blood samples, including flow cytometry, showed no sign of systemic relapse of the pPCL. Consolidating radiotherapy, 24 Gy/12 fractions, was given and an evaluation MRI scan showed no residual mass.

**Figure 2:**
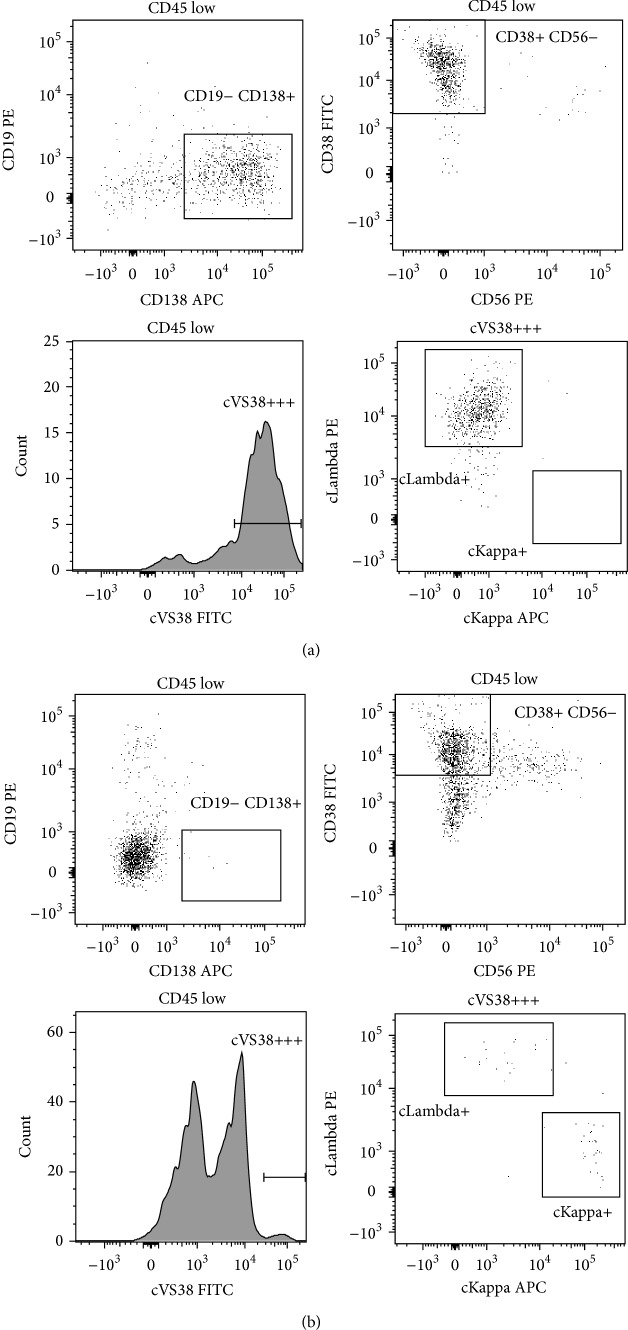
Flow cytometric data at second relapse, February 2012, reveal isolated CNS relapse. (a) CSF with clonal plasma cells with the same phenotype as diagnosis (CD138+, CD38+, and CD56−). (b) Bone marrow aspirate without clonal plasma cells.

In February 2012 the patient presented with weakness of the lower extremities. MRI of the column showed a 10 × 5 mm intramedullary tumour at the level of Th2 and meningeal lesions in the upper cervical region. Cerebrospinal fluid (CSF) had increased number of cells (14 ∗ 10^6^/L) including 60% plasma cells of the same phenotype as the initial circulating plasma cells (Figure 2). The CSF had normal glucose level but increased protein level of 0.59 g/L. Morphological and flow cytometric analyses of the bone marrow and peripheral blood indicated no sign of systemic relapse of the pPCL (Figure 2). The patient was treated with 2 standard cycles of Bortezomib and Dexamethasone and 4 cycles of intrathecal therapy with Methotrexate (10 mg/m^2^), Cytarabine (30 mg/m^2^), and Methylprednisolone (10 mg/m^2^) and subsequently consolidating radiotherapy (21 Gy/7 fractions). Again a complete remission was achieved based on MRI, CSF, and peripheral blood. No reevaluation of bone marrow was done since this second relapse was localized to the CNS. The patient, however, never regained the full function of his legs.

In August 2012 the patient presented with diffuse neurological symptoms and an MRI of the cerebrum showed a tumour of the hypothalamus inaccessible to a biopsy. CSF showed no plasma cells and normal glucose level but increased protein level of 0.77 g/L. However, the diagnosis of a plasmacytoma seemed reasonable and again 2 cycles of Bortezomib and Dexamethasone and 4 cycles of intrathecal therapy with Methotrexate, Cytarabine, and Methylprednisolone were administered. In September 2012 the patient was admitted with septicaemia and general fatigue and treatment was ceased. The patient died shortly afterwards.

## 3. Discussion

This case demonstrates the aggressive nature of pPCL and illustrates the relatively rare involvement of CNS in plasma cell malignancies. The exact prevalence of CNS involvement in pPCL is unknown due to limited cases in the literature. However, previous studies suggest that CNS plasmacytosis is markedly increased in pPCL compared to MM [11, 14]. Also, CNS involvement of MM is associated with high-risk cytogenetics and extramedullary manifestations [14], both of which are seen more frequently in pPCL [1]. In preliminary results from the first larger trial of 40 patients with pPCL two out of 25 responders to induction therapy had meningeal involvement on relapse [18].

Interestingly, most of the previously published cases on CNS relapse of pPCL showed no sign of systemic relapse [10, 12], like in our case. It is well known from the clonal evolutionary theory that one or more clones escape treatment and reemerge in a relapse later on in a disease. Recommended treatment modalities in pPCL as well as MM do not include chemotherapeutics with CNS penetrating effect, which makes the CNS a possible refuge. Thus, we would most likely expect to see more cases of CNS involvement of pPCL as the OS increases with improved treatment strategies.

With this case we would like to point out the importance of investigations including CNS MRI scan and CSF examination in pPCL patients presenting with neurological symptoms.
